# Radiopharmaceuticals heat anti-tumor immunity

**DOI:** 10.7150/thno.79806

**Published:** 2023-01-01

**Authors:** Qi Sun, Jiyuan Li, Zexuan Ding, Zhibo Liu

**Affiliations:** 1 Peking University-Tsinghua University Center for Life Sciences, Academy for Advanced Interdisciplinary Studies, Peking University, Beijing 100871, China; 2 Beijing National Laboratory for Molecular Sciences, Radiochemistry and Radiation Chemistry Key Laboratory of Fundamental Science, College of Chemistry and Molecular Engineering, Peking University, Beijing 100871, China; 3 Changping Laboratory, Beijing 102206, China

**Keywords:** anti-tumor immunity, radiopharmaceutical therapy (RPT), immunogenic cell death (ICD)

## Abstract

Radiopharmaceutical therapy (RPT) has proven to be an effective cancer treatment with minimal toxicity. With several RPT agents approved by FDA, the remarkable potential of this therapy is now being recognized, and the anti-tumor immunity induced by RPT is beginning to be noticed. This review evaluates the potential of RPT for immune activation, including promoting the release of danger associated-molecular pattern molecules that recruit inflammatory cells into the tumor microenvironment, and activating antigen-presenting cells and cytotoxic T cells. We also discuss the progress of combining RPT with immunotherapy to increase efficacy.

## Introduction

Radiopharmaceutical therapy (RPT) is a novel radiotherapy modality defined by the delivery of radionuclides to tumors systemically or locoregionally in a targeted manner, showing strong anti-tumor efficacy 1, 2. Almost all radionuclides used in RPT can be visualized by nuclear medicine based imaging techniques, providing non-invasive visualization of radiopharmaceuticals while ensuring precision delivery 3-6. RPT has made significant progress in the past several years with the development of new radionuclides and vectors and the improvement of labeling efficiencies and targeted property 7-9. These recent developments indicate that RPT is poised to emerge as an effective, safe, and economical therapeutic modality with tremendous potential. Unlike the external irradiation used in traditional radiotherapy, RPT delivers cytotoxic radiation directly to cancer cells or tumor microenvironments systemically or locally 1, 2, 10. The radiation exposure from RPT is continuous, with the unique exponential decay spectrum depending on the characteristics of the radionuclide utilized, consisting of α or β particles, auger electrons, and gamma emissions of varying energies 11-14.

Radiobiology is fundamental to understanding the therapeutic capacity of RPT. There are accumulating investigations into the cytotoxic effects of RPT on tumors, however, direct cytotoxicity is not the only process accounting for tumor destruction. Growing evidence shows that ionizing radiation elicits anti-tumor effects that exceed cell killing, and immune activation plays an important role in response to radiation which contributes to tumor elimination. Radiotherapy could modulate the immunogenicity of tumor cells, and augment innate and adaptive immune responses against tumors, thereby decreasing immunosuppression and potentiating the responsiveness of tumors to radiation both in the tumor microenvironment (TME) and even at a systemic level 15, 16. Irradiated tumor cells undergo a stressful death process, associated with the upregulation of immunomodulatory cell surface molecules, expansion of the cellular peptide pool, and the release of cytokines, which are also known as danger-associated molecular patterns (DAMPs) 17. Then antigen-presenting cells are recruited into TME, subsequently enriching the T‐cell infiltrates, primes, and propagates the pre‐existing or newly infiltrating T cells, inducing anti-tumor effects 18. Notably, current evidence indicates that radiotherapy can also support tumor cell survival via diverse mechanisms 19, 20. The immune response induced by RPT may similar to ionizing radiation, but limited knowledge is available regarding the consequences of targeted RPT on the antitumor immune response.

**Figure 1:**
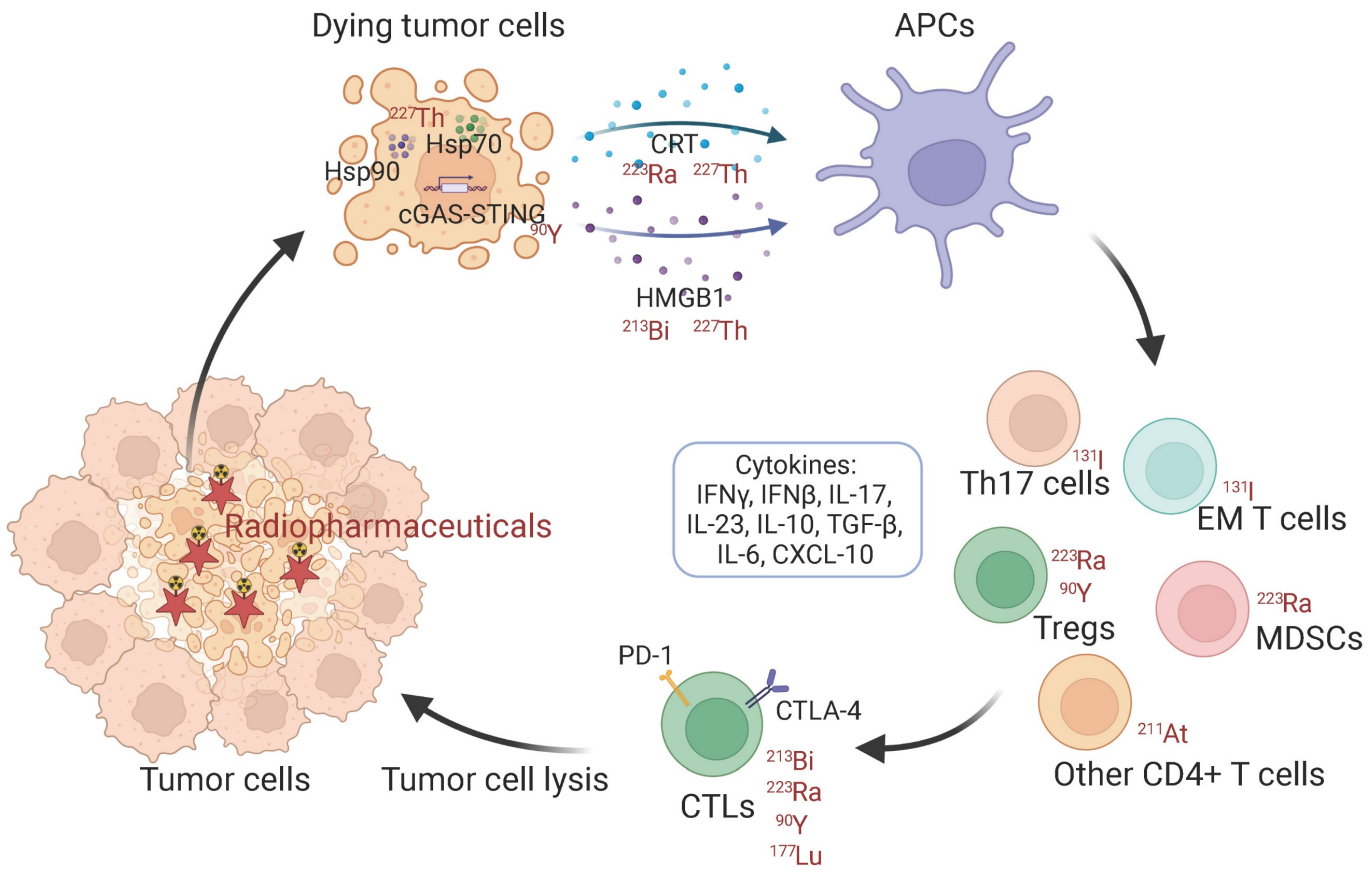
Schematic illustration of immunologic process induced by radiopharmaceuticals. Hsp: heat shock protein. CRT: calreticulin. HMGB1: high mobility group box 1. APCs: antigen-presenting cells. EM T cells: effector memory T cells. MDSCs: myeloid-derived suppressor cells. Treg: regulatory T cell. CTLs: cytotoxic T lymphocytes.

Given that antitumor immunity may help to achieve the ultimate goal of the improvement of the therapeutic efficacy of RPT to cure cancer, there is a need to focus on the RPT-induced antitumor immune response and the synergistic effects with immunotherapy (IT) approaches. In this review, we discussed in detail the immune response activated by radionuclides with different emission properties, and present a brief overview of the interaction between RPT and the immune system (Figure 1). And we provide insight into recent data from pre-clinical and early-phase clinical trials that have investigated RPT in combination with immunotherapy, further highlighting limitations and future challenges.

## Basic immune responses of radiotherapy

Usually, tumor cells can be eliminated by the innate and adaptive immune system leading to complete immunologic eradication of cancer, but a prerequisite for efficient elimination is the recognition of danger signals on tumor cells. It is possible that some cells could progress into an equilibrium phase and begin a chronic tug-of-war with immune system, in this state the immune system is still able to keep these cells under control. However, the final stage of interplay between the immune system and an emergent cancer is that uncontrolled tumor cells successfully escape the immunosurveillance, then grow progressively due to their reduced immunogenicity and the establishment of immunosuppressive tumor microenvironment 21, 22.

Even though this relationship between fundamental process of tumor growth and immune system has been known for a long time, it was once generally believed that there was no direct synergy between radiotherapy-induced local tumor regression and the immune system. Instead, radiotherapy was considered immunosuppressive due to bone marrow and lymphocytes being known to be radiosensitive and may be damaged under systemic irradiation 23.

Nevertheless, recent studies led to a paradigm shift in which radiotherapy can also achieve immunostimulatory effects. There are emerging and convincing hints that a contribution of complex radiotherapy-induced immune activation mechanisms can no longer be neglected, radiation can enhance both the priming and the effector phase of the immune response whether by directly inducing an immunogenic tumor cell death or by altering the tumor microenvironment 24, 25. Undoubtedly, in addition to cell death induction, a productive anti-tumor immune response induced by irradiation is also key to its therapeutic success. Notably, some reports have observed that these immune responses would not only target local tumors but may also treat out-of-field metastases, which has been described as the abscopal effect 26-30.

The main target of irradiation is the induction of DNA damage, abnormal DNA damage repair leading to the deficiency of DNA damage response (DDR) which has recently emerged as an important determinant of tumor immunogenicity 31. DDR caused by radiotherapy could promote the antigenicity and adjuvanticity of targeted tumors. Radiation enhances mutability and genomic instability, increasing the degradation of existing proteins and new peptide production which results in an increase in intracellular peptide pool and thus new tumor-associated antigens (TAAs) 24. These TAAs may be captured by antigen-presenting cells (APCs), which process TAAs into short peptides that are presented on the cell surface that diversifies the antigen presented, leading to an expanded repertoire of tumor-specific CD8+ T cells. After tumor antigen-specific T cells attack the tumor, other new antigens may be released and captured, which is called 'epitope spreading' 32, creating a positive feedback loop and evoking durable and adaptable immune responses against tumors. In addition, the tumor antigens after radiation exposure may vary significantly, depending on the cell types as well as the dose of radiation applied 33. And the changes in the antigenic landscape have not been fully explored.

Meanwhile, activation of cyclic GMP-AMP synthase-stimulator of interferon genes (cGAS-STING) pathway that senses radiation-induced damaged DNA are capable of generating adjuvant activity for enhancing adaptive immune responses to tumor antigens released 34, 35. These new antigens could act as an immune-activating danger signal for antigen-presenting cells (APCs) and thus contribute to adaptive immune system 36.

In particular, the immunogenic cell death (ICD) elicited by radiotherapy also links the DDR to anti-tumor immunity, which refers to cell death modalities that share the propensity to activate an immune response 25, 37. ICD following radiotherapy triggers the emission of a plethora of mediators into the extracellular space as danger signals, which are termed DAMPs. DAMPs represent a large range of molecules and originate from different sources, including extracellular proteins such as fibronectin and biglycan, and intracellular proteins, such as high mobility group box 1 (HMGB1) 38, 39, heat-shock proteins 40, adenosine triphosphate (ATP) 41, 42, calreticulin 43, 44 and Il-1α 45, 46 that only released following stress or cell death. These DAMPs are recognized by macrophages and dendritic cells (DCs) triggered by different pathways including Toll-like receptors (TLRs), induce their maturation and promote cross-presentation of tumor antigens, stimulate the release of cytokines including IL-1β, IL-23, and CXCL-10 e.g., upregulate the co-stimulatory molecules, that in turn is conducive for the infiltration and chemotaxis of immune effectors. Activated natural killer cells (NKs) and cytotoxic T lymphocytes (CTLs, which are CD8+ T cells) possess the ability to kill tumor cells with the help of CD4+ T cells 46-49. IFN-γ and TNF-α, the CTL signature cytokines, also have anti-tumor mediating properties 50, 51. In addition, radiotherapy can upregulate multiple pro-inflammatory cytokines, including tumor necrosis factor-α (TNF-α), IL-6, IL-8, IL-1α, and IL-1β, to induce an acute inflammatory reaction in tumors 46.

Consequently, accumulating evidence suggests that radiotherapy could kill tumor cells via induction of anti-tumor immune responses by acting as in situ vaccine. However, the dialogue between radiotherapy and immune responses is mostly unknown. Radiotherapy sometimes also plays a role in immunosuppression 52-54. While extensive research concerning DNA damage and ICD following radiotherapy has been carried out, there is still a lack of data in pre-clinical and clinical studies about its effects on the immune system, with special regard to the impact of different radiation regiments.

Within radiation, RPT with high LET differs from X-ray radiation but shares common features of DNA damage 1, 55-57. It is important to understand whether the impacts of RPT on the immune system follow the common characteristics of radiotherapy or whether there are specific response signaling pathways, which could guide the clinical treatment of RPT in combination with immunotherapy to improve anti-tumor efficacy. Data about the influence of RPT concepts on immunological consequences are scarce and not conclusive, and present knowledge will be reviewed in the following. Radionuclides are used to deliver radiation with different emission properties: α-particles or β-particles 1. Here we present a summary of radiopharmaceutical-induced anti-tumor immune activation according to the types of radiation and the radionuclides (Table 1).

## α-emitting radiopharmaceuticals propagate antitumor immunity

### Physical properties of α-emitting radionuclides

An α-particle is a positively charged helium ion (2p, 2n, +2e) that is emitted from the nucleus of an unstable atom. Alpha particles are energetic with a typical kinetic energy of 5-8 MeV traveling at 5% of speed of light losing a large amount of energy (high linear energy transfer of about 100 keV/μm) in a short path of 50 to 80 μm in view of their electric charge and relatively large mass 58, 59. Several studies have been performed to describe α emitter radiobiology and cell death mechanisms induced after α irradiation. Like other high linear energy transfer particles, α emitters induce more DNA double-strand breaks than γ or X-rays and provoke a cell cycle arrest in the G2 phase that is more marked than with γ rays 60-63. Furthermore, radiobiologic effects associated with α radionuclides are advantageously less sensitive to dose rate, hypoxia, and cell cycle distribution than β particles or γ rays 64, 65.

Alpha-particle emitting radionuclides have also been applied for RPT, including ^211^At, ^213^Bi, ^223^Ra, ^225^Ac, ^227^Th e.g., 1. The alpha emitters have tended to show particular promise and there is substantial interest in further developing these agents for therapy of cancers that are particularly difficult to treat. When properly targeted to disease sites, α particles may lead to cellular death through catastrophic clustered DSBs in the nucleus which is difficult to repair and threatens genome integrity, then may further influence multiple aspects of tumor immunogenicity and have immunomodulatory effects in the tumor microenvironment, making them compelling for RPT.

### Immunologic effects of α-emitting radionuclides

#### ^213^Bi

The first investigation to analyze propagating antitumor immunity of α particles was undertaken by J. B. Gorin et al (2014) 66. They studied the immunogenicity of ^213^Bi-BSA on mouse MC38 adenocarcinoma. ^213^Bi emits both α (~92.7%) and β (~7.3%) particles with a relatively short half-life of 46 minutes. Irradiated MC38 cells were injected into immunocompetent C57Bl/6 mice in a vaccination approach, and the overall survival increased 2 months after vaccination from 16% in the control group to 88%. The lasting protective effect was indicated by tumor transplantation rechallenge, and the immunization with irradiated MC38 failed in nude mice. These results indicated that even long after vaccination, irradiating cancer cells with ^213^Bi can stimulate adaptive immunity mediated by tumor-specific T cells. Regarding the possible molecular mechanisms, they also showed that ^213^Bi-BSA of MC38 cells in vitro induced the release of DAMP, such as heat shock protein 70 (Hsp70) and HMGB1 through ELISA performed on a conditioned medium. In addition, the activation of co-cultured bone marrow-derived DCs was observed through the upregulation of costimulatory molecules (CD40, CD80, and CD86).

#### ^223^Ra

^223^RaCl_2_ is the first approved targeted α-emitting radiopharmaceuticals by the U.S. Food and Drug Administration (FDA). It has been used in clinics since 2013 to treat patients with metastatic castration-resistant prostate cancer (mCRPC), but still limited preclinical and clinical studies of antitumor immune responses induced by ^223^Ra were discussed. ^223^Ra is a calcium mimetic and naturally targets the bone hydroxyapatite (Ca_10_(PO_4_)_6_(OH)_2_) matrix 67-70. ^223^Ra decays to stable ^207^Pb with an emitted decay energy distribution of 93.5% α particle, <3.6% β- particle, and <1.1% γ radiation with a half-life of 11.43 days 71.

In 2016, A. S. Malamas et al. reported the immunogenic modulation in tumor cells (human prostate, breast, and lung carcinoma cells) exposed to sublethal doses of ^223^Ra in vitro 72.^ 223^Ra significantly enhanced T cell-mediated lysis of each tumor type (MDA-MB-231, ZR75-1, LNCaP, PC3, H1703, and H441 cells) by CD8+ CTLs specific for MUC-1, brachyury, and CEA tumor antigens. Immunofluorescence analysis revealed that the increase in CTL killing was accompanied by augmented protein expression of MHC-I and calreticulin. Kim JW et al. discussed immune responses of circulating peripheral blood mononuclear cells (PBMCs) of bone metastatic castration-resistant prostate cancer (mCRPC) patients after ^223^Ra radiation, fifteen patients received a course of ^223^Ra 50 kBq/kg 73. PD-1 expressing EM CD8+ T cells decreased after one ^223^Ra treatment from 20.6% to 14.6%, while no significant change was observed in the frequencies of CD27, CD28, or CTLA4-expressing T cells. However, no significant change in the overall frequencies of CD8+ T cells including naïve, central memory and effector memory (EM) cells, and also the frequencies of CD8+ T cells producing IFN-γ, TNF-α, and IL-13. Another similar case was reported, a total of 35 mCRPC patients were screened with ^223^Ra administered intravenously every four weeks at a dose of 55 kBq/kg (a maximum of six injections) 74. H. A. Jeroen et al. observed a decrease in absolute lymphocyte counts and an increase in the proportion of T cells that expressed costimulatory (ICOS) or inhibitory (TIM-3, PD-L1, and PD-1) checkpoint molecules. All memory and effector phenotypes within the CD4+ and the CD8+ subset appeared stable throughout ^223^Ra therapy. Moreover, the fraction of two immunosuppressive subsets (the regulatory T cells and the monocytic MDSCs) increased throughout the treatment which was contrary to Kim's results. They hypothesized that Tregs, M-MDSCs, and checkpoint-expressing T cells may be the reflection of the migration of (non-exhausted) effector T cells into the tumor.

Abscopal effects also be occurring with ^223^Ra-dichloride therapy. Kwee et al. reported observations in 2 patients that suggested ^223^RaCl_2_ abscopal effect with resultant favorable response in untargeted soft tissue disease 75. Concomitant increases in plasma interleukin 6 were detected, suggesting that the response may be mediated by immune activation.

#### ^227^Th

In 2019, Hagemann et al. presented the preclinical evaluation of a mesothelin (MSLN)-targeted thorium-227 conjugate, BAY 2287411, for the treatment of MSLN-expressing tumors 76. On OVCAR-3 cells, they found that BAY 2287411 was able to upregulate the DAMPs in vitro, including calreticulin, HSP70, HSP90, and HMGB1 detected. However, additional studies are needed in mouse animal models to evaluate the potential immunostimulatory effects of BAY 2287411.

#### ^211^At

In 2021, Hannah Dabagian et al. observed the enhanced recruitment of macrophages and CD4+ T cells to tumors treated with [^211^At]MM4 assessed by histopathology of a poor mouse responder *in vivo* 77. But CD4+ T cells contain immunosuppressive T-regulatory cells, so more evidence is needed to demonstrate the pro-inflammatory processes. Jiajia Zhang et al. designed the ^211^At labeled Mn-based radiosensitizer (^211^At-ATE-MnO_2_-BSA), which increased the proportion of CD80+CD86+ DCs on mice bearing CT26 tumors, compared to the group of free ^211^At treatment or MnO_2_-BSA treatment 78.

## β-emitting radiopharmaceuticals propagate antitumor immunity

### Physical properties of β-emitting radionuclides

β-particles are electrons emitted from the nucleus, which are the most frequently used emission type for RPT agents. β-particle have a longer path length in tissue (1-10 mm), but a lower LET (0.2 keV/μM), which leads to less complex cell damage and is more readily repaired 79, 80. The β-particle emitters yttrium-90, and iodine-131, samarium-153, and lutetium-177, have been introduced and are commonly used 1.

### Immunologic effects of β-emitting radionuclides

#### ^90^Y

Mala Chakraborty et al. studied ^90^Y-labeled COL-1, using CEA-transgenic mice transplanted with MC38-CEA+ tumor cells. COL-1 (a murine IgG2a) is a murine antibody specific for CEA (human carcinoembryonic antigen). They found that cell-surface expression of Fas was upregulated in irradiated than in nonirradiated MC38-CEA+ tumor cells, and peaked at the treatment of 50 and 100 μCi ^90^Y-labeled COL-1. Through MC38-CEA-DN1 tumors (Fas nonfunctional) models, they confirmed that increased survival treatment with ^90^Y-labeled COL-1 was mediated by engagement of the Fas/Fas ligand pathway. However, in MC38 cells, the expression of Fas was not increased, showing the importance of retaining radiolabeled antibodies in the phenotypic changes of tumor cells 81.

NM600 is an alkylphosphocholine analog that exhibits preferential uptake and accumulation in nearly all tumor types. ^90^Y-NM600 is delivered to tumor microenvironments (TME) for tumor therapeutic, and three groups studied the immune effect of ^90^Y-NM600 82. Justin Jagodinsky et al. treated B16 or MOC2 tumor cells by ^90^Y-NM600 (140 µCi), and splenocytes were added three days after irradiation. By flow cytometry one day later, they found when co-cultured with ^90^Y-NM600-treated tumor cells, live CD4 and CD8 cells number and the expression of IFN-γ of CD8+ T cells increased, at the same time, CTLA-4 on T cells, an inhibitory immune signal, also increased compared to those co-cultured with untreated control tumor cells. In addition, using STING KO cells, they determined that the activation of IFN-γ production is independent of STING pathway 83. Reinier Hernandez et al. established mice bearing T-cell NHL tumors treated with ^90^Y-NM600 (9.25 MBq) which experienced tumor growth inhibition and extended survival with immune memory. By immunohistochemistry staining, increased CD8+ T cells and decreased Foxp3+ regulatory T cells at day 6 were discovered. And tumors did not grow 10 days after re-inoculation of tumor cells. Besides, they transplanted adoptive T cells from radiated mice to Rag2 KO (immunocompromised) mice, the tumor tissues of Rag2 KO mice achieved sustained complete response, suggesting the tumor suppression was dependent on T-cells activation 82. Furthermore, Ravi Patel et al. investigated immunomodulatory effects of ^90^Y-NM600 *in vivo* by C57Bl/6 mice bearing B78 flank tumors treated with 50 μCi ^90^Y-NM600. They observed that the ratio of effector T cells (CD8+) to suppressor Tregs (CD4+CD25+FOXP3+), expression of Vcam1, Fas, and IFNβ, increased at day 1 after treatment with a modest decrease in Il6. Activation of the cGAS/STING pathway was found to be required for antitumor efficacy by STING KO model. Besides, innate myeloid (CD11b+) and NK cells were significantly increased on day 7 after radiation 84.

However, before and after ^90^Y radioembolization, low levels of tumor‐infiltrating lymphocyte (TIL) infiltration were observed in tumor tissue of patients with metastatic colorectal cancer, suggesting the lack of immunomodulatory responses to ^90^Y radioembolization in this clinical pilot feasibility study 85.

#### ^131^I

^131^I radioiodine has been used for many years to treat thyroid cancers 86. Previous studies have revealed that T helper cells are an important immunological mechanism in the pathogenesis of differentiated thyroid cancer (DTC) 87. Lixia Zhang et al. examined the distribution of Th17, Tc17, and Treg cells in patients with DTC before and after ^131^I therapy, they discovered that at 90 days following ^131^I therapy, the numbers of Th17, Tc17, and Treg cells, as well as the levels of related cytokines (IL-17, IL-23, IL-10, and TGF-β1) decreased and returned to the similar values as detected in healthy control patients 88.

Yu Chao et al. designed an *in situ* gelation strategy to trap a ^131^I within sodium alginate (ALG), catalase (Cat), and CpG oligonucleotide, ^131^I-Cat/CpG/ALG. In the ^131^I-Cat-CpG/ALG treatment group, more effector memory T cells (TEM), and higher serum levels of TNF-α and IFN-γ were observed following tumor rechallenging, indicating long-term protection of immunological memory induced by ^131^I-Cat-CpG/ALG 89. It is worth noting that substances other than ^131^I in the hydrogel may play a role in immune activation, so we must be cautious that the immunity caused by ^131^I-Cat-CpG/ALG may not completely represent the effect of ^131^I.

#### ^153^Sm

^153^Sm lexidronam is a chelated complex of a radioisotope of the element samarium with EDTMP that binds avidly to hydroxyapatite in bone, has been approved for the treatment of pain associated with bone cancer in 1997 90. Mala Chakraborty et al. explored the phenotype of tumor cells and T cell-mediated killing. Using 10 human tumor cell lines exposed to ^153^Sm-EDTMP, at least two of the five surface molecules (Fas, CEA, MUC-1, MHC class I, and ICAM-1) on each cell line were upregulated. In addition, treatment of LNCaP cells with ^153^Sm-EDTMP functionally increases antigen-specific CTL-mediated killing, suggesting an induced immune response 91.

#### ^177^Lu

In 2018, FDA approved lutetium ^177^Lu-DOTATATE (Lutathera) for the treatment of somatostatin receptor-positive gastroenteropancreatic neuroendocrine tumors (GEP-NETs). And ^177^Lu-PSMA-617 (Pluctivo) is now approved in 2022 for the treatment of patients with prostate-specific membrane antigen (PSMA)-positive metastatic castration-resistant prostate cancer (mCRPC) who have previously been treated with an androgen-receptor pathway inhibitor and taxane-based chemotherapy. It is promising to understand the immune response induced by ^177^Lu.

^177^Lu-DOTATATE is a type of peptide receptor radionuclide therapy (PRRT) for the treatment of neuroendocrine tumor (NET) patients approved by the FDA approval in 2018 92. By using NCI-H727 cells xenograft model of NETs, Yin W et al. showed that ^177^Lu-DOTATATE PRRT led to increased infiltration of CD86+ cells and CD49b+/FasL+ NK cells in tumor tissues, which is capable of tumor killing 93. Haojun Chen et al. treated MC38 tumor-bearing C57BL/6 mice with 18.5 MBq ^177^Lu-EB-RGD, which contained Arg-Gly-Asp (RGD) sequence specifically targets the cell surface receptor integrin αvß3. On days 4 and 7, the percentage of CD45+/PD-L1+ and CD11b+/PD-L1+ cells increased at least twofold, indicating that ^177^Lu-EB-RGD led to an acute increase in PD-L1 expression on T cells 94. Additionally, ^177^Lu-DOTA-diZD induced immune response was studied 95, which is a high-affinity vascular endothelial growth factor receptor (VEGFR)-targeted agent labeled with ^177^Lu by 1,4,7,10-tetraazacyclododecane-1,4,7,10-tetraacetic acid (DOTA) chelator. In 4T1-bearing mice treated with ^177^Lu-DOTA-diZD, the population of CD4+ and CD8+ cells increased, suggesting that ^177^Lu-DOTA-diZD modulates the tumor microenvironment.

#### ^188^Re

Treated with 1.5 mCi ^188^Re-6D2 which binds to melanin, the tumors of A2058 human melanoma xenograft model were suppressed and the pronounced presence of complement C3 was discovered, which was able to activate the host immune system 96.

#### ^18^F

In addition, positron emission tomography (PET) imaging tracer 2-[^18^F]FDG may also be an immunomodulator. After co-incubation with 2-[^18^F]FDG (1.85 MBq/mL), the proportions of PD-L1+ cells of CT26, MC38, 4T1, and B16F10 tumor cells were significantly increased, which was induced by DNA damage via STAT1/3-IRF1 pathway. And the upregulation of PD-L1 expression has also been demonstrated in CT26 and MC 38 tumor-bearing mice models 97, which may make tumor cells more sensitive to anti-PD-L1 antibody treatment.

#### ^64^Cu

64Cu decays in three ways: β+ (17%, Emax = 655 KeV), β- (39%, Emax = 573 KeV) and electron capture (44%) 98. ^64^Cu-based radioligands have been developed for PET imaging, while ^64^Cu might be a potential therapeutic radionuclide. Similar to 2-[^18^F]FDG, 370 kBq of ^64^Cu-DOTA-EB-cRGDfK also upregulated PD-L1 expression in CT26 and MC38 cells 99.

**Figure 2:**
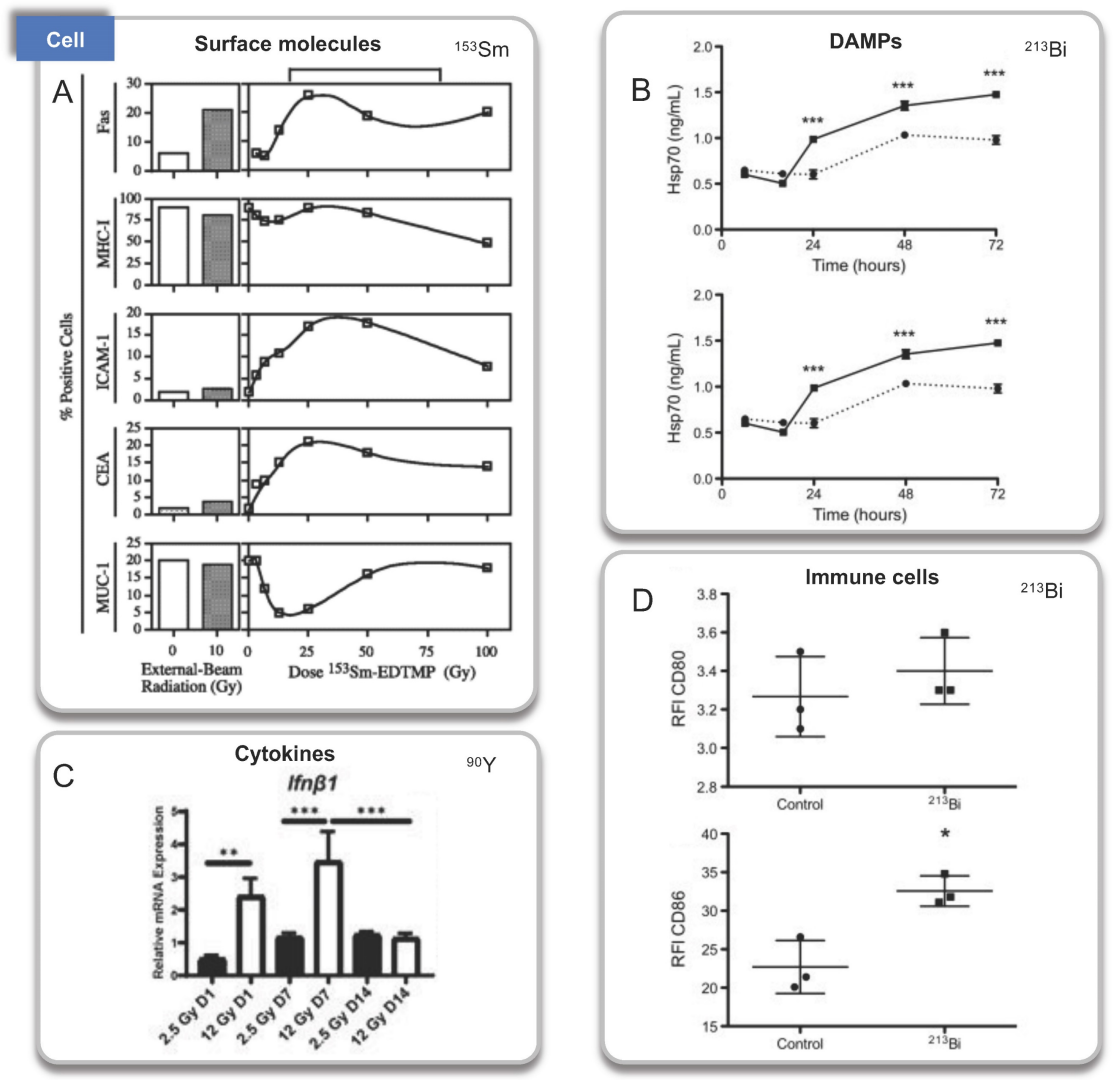
Immunologic processes activated by RPT confirmed by cell experiments. A. Surface molecules, ^153^Sm; B. DAMPs, ^213^Bi; C: Cytokines, ^90^Y. D. Immune cells, ^213^Bi. Panel A is adapted with permission from 91, copyright 2021 by the American Association for Cancer Research. Panel B and D is adapted with permission from 66, copyright 2021 Elsevier, Inc. Panel C from 83.

**Figure 3:**
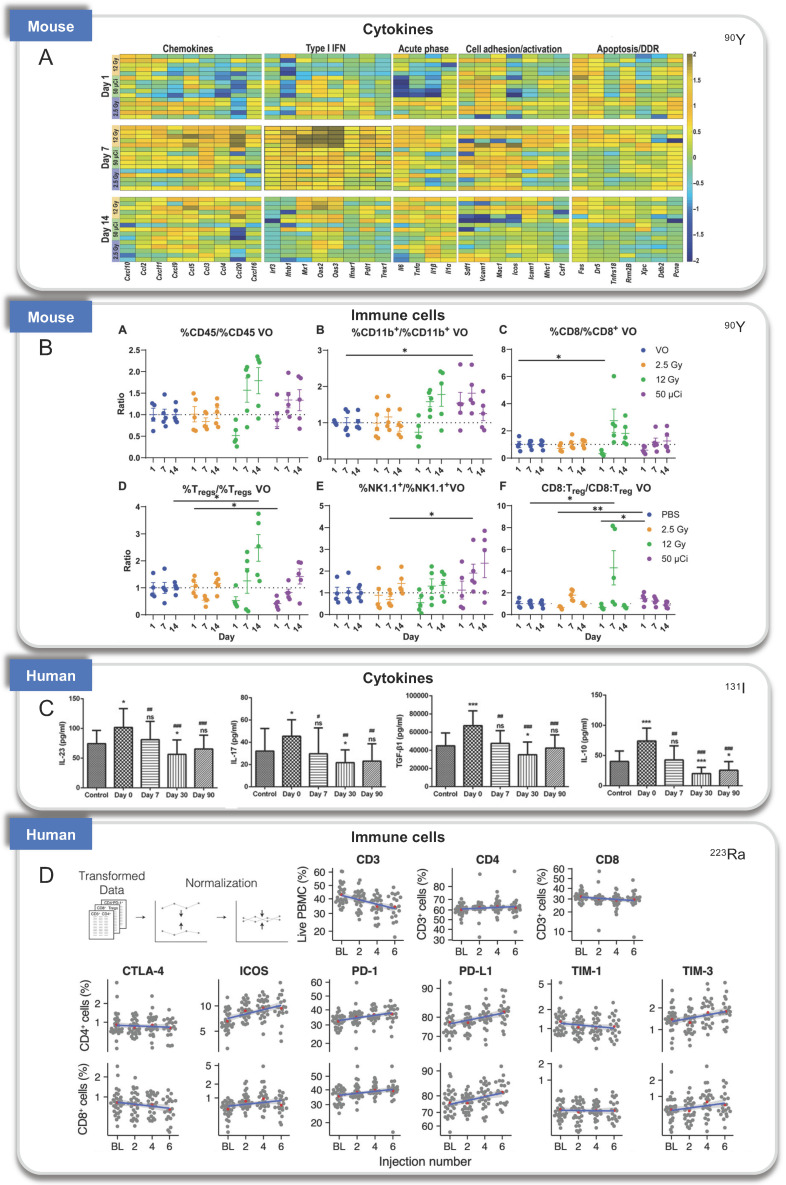
Immunologic processes activated by RPT found in preclinical and clinical studies. (A-B). A. Cytokines and B. immune cells induced by ^90^Y in mouse models; C. Cytokines induced by ^131^I in patients; D: Infiltration of immune cells treated by ^223^Ra in patients. Panel A and B is adapted with permission from 84, copyright 2021 American Association for the Advancement of Science. Panel C is adapted with permission from 88, copyright Spandidos Publications 2021. Panel D is adapted with permission from 131, copyright 2007 - 2021 Frontiers Media S.A.

**Figure 4:**
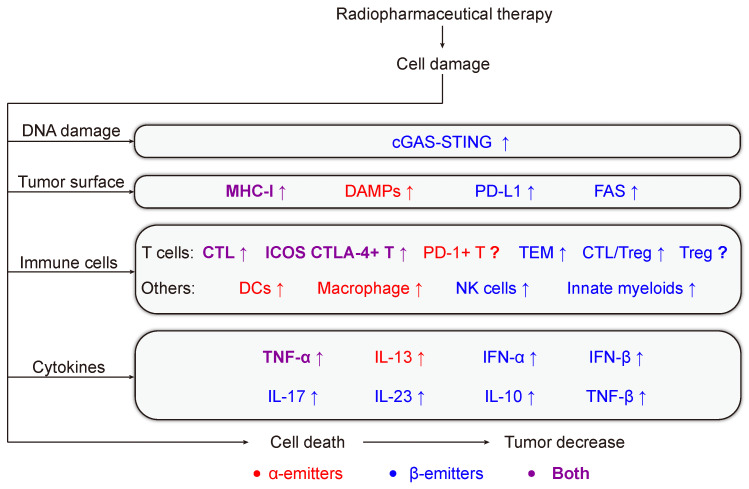
RPT-induced anti-tumor immune response. RPT causes DNA damage, induces the expression of tumor antigens, recruits immune cells, enhances the secretion of cytokines in the tumor environment, and kills tumor cells. Red and blue present α-emitters or β-emitters respectively. Purple represents molecules, signaling pathways, or cells that are involved in common.

See Figure 2-4 and Table 2 for a summary of the RPT-induced effects based on the activated immune process.

## Combination of immunotherapy with RPT to increase anti-tumor efficacy

### Pre-clinical studies with combined strategies to enhance tumor killing effects

Although according to the above summary, nuclear medicine can activate the immune system to enhance anti-tumor effects, there are not many reports or clinical data. Additionally, only a small percentage of patients achieve complete response through just radiotherapy, a common point seems to be the fact that radiation probably can only amplify a pro-immunogenic phenotype and can hardly change by itself a net immune-suppressing environment into an immune-stimulating one. Thus, future research could focus on defining optimal RPT protocols on one hand, and optimal combination approaches on the other hand to achieve greater therapeutic effects than the respective monotherapies and lower dosages or numbers of cycles required, in turn, reducing unwanted toxicities. Several preclinical investigations have suggested the combination of radiation and immune checkpoint inhibitors (ICI) promotes response and immunity 100-102. To improve the anti-tumor effect of nuclear medicine and better take advantage of nuclear medicine in inducing an anti-tumor immune response, radioimmunotherapy has proven to be a useful tool.

At present, it is clinically applied immunotherapy target ICI with programmed death 1 (PD-1) 103 and cytotoxic T-lymphocyte-associated antigen 4 (CTLA-4) 104, 105, expressed in T cell surface negative direction The T cell function is regulated so that the tumor tissue is immunized. The inhibitors of these two targets can activate the immune system to generate significant improvements in disease outcomes for various cancers. PD-1 inhibitors mainly act on the effect phase of T cells. T cells will express PD-1 after being activated, and tumors evade the monitors of immune systems by expressing PD-L1 which is the ligand of PD-1, thus the anti-tumor activities of T cells are often inhibited 103. The PD-1 inhibitor can block the binding of PD-L1 and PD-1, re-release the anti-tumor capacity of tumor suppression. Nivolumab 106 and pembrolizumab 107, 108, Both PD-1 Inhibitors, have been approved to treat patients with advanced or metastatic melanoma and patients with metastatic, refractory non-small cell lung cancer. PD-L1 inhibitors Atezolizumab 109, Durvalumab 110 and Avelumab 111 have been approved to treat non-small cell lung cancer, locally advanced or metastatic urinary tract cancer, Merkel cell carcinoma. The CTLA-4 inhibitor mainly acts on the early development stage of T cells, by combining CTLA-4 on the surface of T cells, inhibiting the antigen presenting cells on T cell function, indirectly promoting the activation and proliferation of T cells. Ipilimumab, an inhibitor of CTLA-4, has been approved for the treatment of advanced or unresectable melanoma 112, 113. Although immune checkpoint inhibitor treatment may be effective initially, many patients will eventually relapse and develop tumor progression, and only part of the patients can achieve complete response 114.

Thus, the use of RPT activation of immune systems while inhibiting tumor immune escape may be more efficient than a separate treatment to effectively inhibit tumor growth. As of now, there have been limited pre-clinical attempts to improve RPT outcomes through combinations with immunotherapy. Here, we present a review of the combination strategies of TRT with immunotherapy reported in the literature to date.

Johannes Czernin et al. found a synergistic anti-tumor effect used in nuclear drugs and PD-1 inhibitors. C57BL/6-mice bearing syngeneic RM1-PGLS tumors were treated with 30 kBq ^225^Ac-PSMA617 (on day 12), 10 mg/kg Anti-PD-1 Antibody (on days 13, 16, 20, and 23), or Both. Combining PSMA-RPT and anti-PD-1 significantly improved disease control compared with either monotherapy. Time to progression was extended to 47.5 days (isotype control, 25 days; ^225^Ac-PSMA617, 30 days; Anti-PD-1, 33.5 days), and survival to 51.5 days (isotype control, 28 days; ^225^Ac-PSMA617, 32 days; Anti-PD-1, 37 days) 115.

Hannah Dabagian et al. investigated the effects of 36 MBq/kg [^211^At]MM4 administered on day 11 in combination with 200 μg anti-PD-1 administered on days 8, 11, and 14 in a syngeneic mouse model of glioblastoma using GL26 glioblastoma cells in C57BL/6J mice. The average best tumor responses for combination, anti-PD-1, and [^211^At]MM4 were 100%, 83.6%, and 58.2% decrease in tumor volume, respectively. Average progression free intervals for combination, anti-PD-1, [^211^At]MM4, and control groups was 65, 36.4, 23.2, and 3 days, respectively. The percentages of disease-free mice at the end of the study for combination and anti-PD-1 were 100% and 60%, while [^211^At]MM4 and control groups were both 0%. In summary, combination therapy was more effective than either single agent in all response categories analyzed, highlighting the potential for PARP targeted alpha-therapy to enhance PD-1 immune-checkpoint blockade 77.

Patrycja Guzik and others explored the anti-tumor treatment of ^177^Lu nuclear medicine combined with an anti-CTLA-4 antibody. NF9006 tumor-bearing mice received ^177^Lu-DOTA-folate (5 MBq; 3.5 Gy absorbed tumor dose), anti-CTLA-4 antibody (3 × 200 μg), or both agents. They found ^177^Lu-DOTA-folate only or ICI only had only a minor effect on tumor growth and did not increase the median survival time (23 days and 19 days, respectively) as compared with untreated controls (12 days). However, tumors treated with combination therapy decreased and median survival time of mice increased (> 70 days). Thus, the application of ^177^Lu with anti-CTLA-4 immunotherapy had a positive effect on the anti-tumor efficiency 116.

Since PD-1/PD-L1 and CTLA-4 possessed different mechanisms of action, the combination therapy using these two inhibitors has also been studied and applied 117, 118. Scientists also explored the anti-tumor effects of nuclear medicine cooperated with these two inhibitors at the same time. They reported the anti-tumor effect studies of 30 MBq ^177^Lu-LLP2A treatment, alone and combined with immune checkpoint inhibitors (200 μg anti-PD-1, anti-PD-L1, and anti-CTLA-4 antibodies on days 9, 12, and 15 after tumor cell injection), in B16F10 tumor-bearing mice. They discovered that the survival of RPT alone was comparable to the dual-ICI anti-PD-1 + anti-CTLA-4 or anti-PD-L1 + anti -CTLA-4, whereas RPT + ICIs significantly enhanced survival. At the same time, TUNEL staining also indicated more apoptosis signals in RPT + ICI groups 119.

In addition to the combination strategy, radioisotope-labeled monoclonal antibodies which target immune checkpoints such as PD-L1 may also be effective cancer treatment strategies, because immune checkpoint-associated antibodies linked to cytotoxic nuclides not only have the function of immune modulation as an ICI, but only can selectively bind tumor antigens and release cytotoxic radiation.^ 213^Bi-anti-hPD-L1 mAb was developed by Marisa Capitao et al 120. Using M113^PD-L1+^ melanoma xenograft model, delayed tumor growth was observed in mice treated with 125 kBq/g ^213^Bi-Anti-hPD-L1 mAb, while no significant change was found in PD-L1 negative M113^WT^ melanoma xenograft model with the same treatment. Improving tumor targeting is a big challenge for designing radioisotope-labeled monoclonal antibodies, with high tumor uptake, long tumor retention and low uptake in other major organs. Jingyun Ren et al. screened the antibody by systematic PET imaging study and labeled it with the ^177^Lu for RIT, denoted as ^177^Lu-DOTA-Y003 121. With the treatment of 11.1 MBq of ^177^Lu-DOTA-Y003, slower tumor growth was shown in MC38-bearing mouse model than the control group, and the survival was prolonged. Although single ^131^I-αPD-L1 alone showed limited tumor suppression developed by Xuejun Wen et al., ^131^I-αPD-L1 could induce PD-L1 expression and further increase the uptake of αPD-L1 mAb in CT26 and MC38 tumors 122. Moreover, the combination treatment of 11.1 MBq of ^131^I-αPD-L1 + 200 μg of αPD-L1 mAb prolonged the survival of mice, of note, cures half of the tumor-bearing mice.

Altogether, these data exhibited that radioisotope-labeled immune checkpoint antibodies have the potential to enhance the efficacy of cancer immunotherapy. Furthermore, since some RPTs have been shown to potentially upregulate PD-L1 expression, this phenomenon could be exploited for the combination therapy in an appropriate time window.

### Harnessing RT to Improve Responses to Immunotherapy

**Figure 5:**
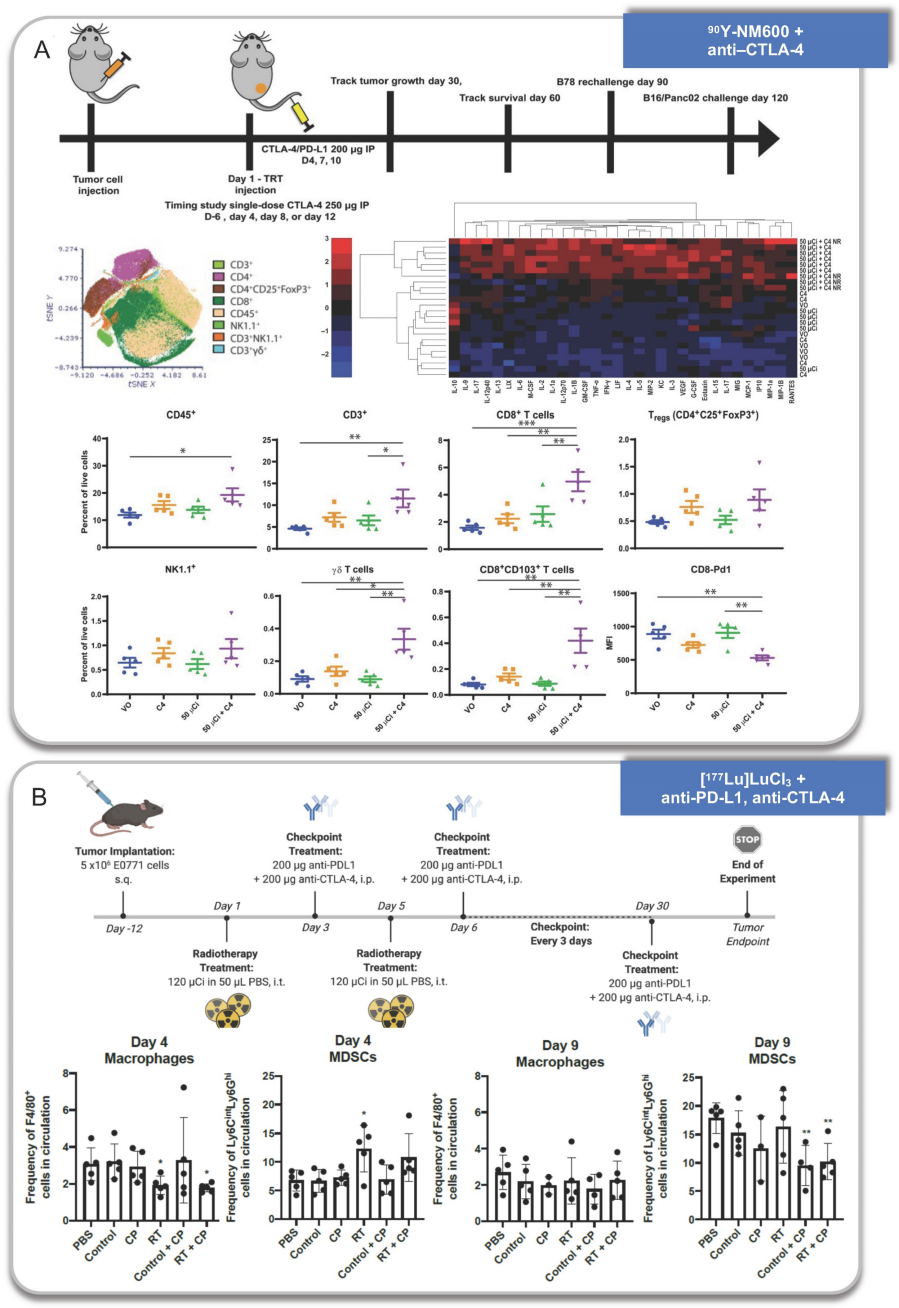
Anti-tumor immune response activated by RPT combined with immunotherapy. A. ^90^Y-NM600 combined with anti-CTLA-4 therapy. B. [^177^Lu]LuCl_3_ combined with anti-PD-L1 and anti-CTLA-4 therapy. Panel A is adapted with permission from 84, copyright 2021 American Association for the Advancement of Science. Panel B is adapted with permission from 132, copyright 1996-2021 MDPI (Basel, Switzerland).

Some works have also helped to explain why the combination strategy is better than monotherapy in the terms of anti-tumor efficacy (Figure 5).

Jiajia Zhang et al. proved that the tumor growths were inhibited in ^211^At-ATE-MnO_2_-BSA group, while ^211^At-ATE-MnO_2_-BSA plus anti-PD-L1 treatment exhibited a prolonged survival rate. In the model of distal tumors, the intratumoral CD8+ T cells increased upon the treatment of ^211^At-ATE-MnO_2_-BSA plus anti-PD-L1 with no obvious changes of Tregs. Also, the level of TNF-α and IFN-γ increased by the synergistic treatment 78. In addition, after 28 days of combinational treatment, the secondary tumors were inoculated on the opposite side of the initial tumors, and found that the proportion of effector memory CD4+ T and CD8+ T cells increased in spleens, suggesting the generation of immune memory.

Xuejun Wen et al. proved that 2-[^18^F]FDG treatment could upregulate the expression of PD-L1 on MC38 tumors, and the combination of 2-[^18^F]FDG (37 MBq) and anti-PD-L1 mAb improved the anti-tumor efficacy and prolonged the overall survival 97. Upon treatment, the increase in the infiltration of the effector memory T (TEM) cells in the spleen was observed, suggesting the enhancement of immunologic memory. Additionally, the numbers of intratumoral CD4+ Th1 (which helps the macrophage or CD8+ T cells mediated immunity) and CD8+ CTLs increased with M1-like macrophages infiltration following the combination treatment, while immunosuppressive regulatory T cells (Treg) and M2-like macrophages showed a decrease. Besides, the proinflammatory cytokines including TNFa, IFNγ, and IL6 increased in serum, and maintained for at least 7 days.

^64^Cu-DOTA-EB-cRGDfK may also increase the expression of PD-L1 in MC38 tumors, and the sequential treatment group of MC38-bearing mice with ^64^Cu-DER (18.5 MBq) followed by αPD-L1 mAb (10 mg/kg) with a 4 h interval displayed complete cure within 30 days 99. Moreover, in the infiltration of CD8+ CTLs (CD45+CD8+IFN-γ+) and CD4+ Th1 (CD45+CD4+IFN-γ+) cells in tumors, the ratios of CD8+ CTL/Treg and CD4+ Teff (CD4+ T effector cell)/Treg in tumors, and the proportion of TEM cells (CD8+CD44^high^CD62L^low^ and CD4+CD44^high^CD62L^low^) in spleen increased 7 days after the combination treatment.

Haojun Chen et al. discovered in MC38 bearing C57BL/6 mice, ^177^Lu-EB-RGD (18.5 MBq) combined with anti-PD-L1 antibody (10 mg/kg) synergistically enhances anti-tumor immunity by stimulating CD8+ T cell infiltration, improving tumor control, overall survival and protecting against tumor rechallenge. Besides, in both concurrent (day 4 and 7) or sequential (day 14) combined therapy, the level of CD8+ T cells is higher than that in mice treated 94.

Ravi Patel et al. observed increased survival with the combination of 50 or 100 μCi ^90^Y-NM600 and anti-CTLA-4, and the combination strategy induced distant tumor response in B87 tumor models. They found CD45+ immune cells, CD3+ T cells, CD8+ effector T cells, CD8+ CD103+ tissue resident effector memory T cells, and γδ T cells increased and PD-1 decreased which suggested reduced immune exhaustion at day 25 after treatment. Meanwhile, most cytokines also increased in the TME, particularly in responding mice. One exception was IL-10, which was reduced in the TME, consistent with a reduction of infiltrating Tregs or suppressive monocyte populations. They further found tumor-infiltrating lymphocytes (TILs) isolated from combination-treated animals produced more IFN-γ upon stimulation. More importantly, an increase in clonal expansion of T cells was also observed through deep TCR-β sequencing 84.

Based on Yu Chao's experimental design, ^131^I was used to label Cat, and combined with CTLA-4 inhibitors to explore the remote effects and mechanisms 89. After local injection of the ^131^I-Cat/ALG hybrid solution into tumors, Ca^2+^ triggers rapid gelation of ALG, and by introducing immune-adjuvant CpG, local treatment with a mixture of ^131^I-Cat/CpG/ALG could trigger stronger systemic anti-tumor immune responses. They used ^131^I-Cat/CpG/ALG hybrid solution plus CTLA-4 checkpoint-blockade therapy in distant CT26 tumors, a remarkable synergistic effect to eliminate distant metastatic tumors was found, with increased CD8+ CTL infiltration and decreased Treg cells in the distant tumors. At 20 days post different treatments, TNF-α and IFN-γ increased in mice sera which play crucial roles in the cytotoxic functions of CTLs. And this combination therapy also provided long-term immune memory protection for treated mice. Through T cell blocking experiments using anti-CD4 and anti-CD8 antibodies, both CD4+ and CD8+ T cells were indicated important to the abscopal effect.

In addition to using immune checkpoint inhibitors, Mala Chakraborty and others have also tried recombinant anticancer vaccine, they treated CEA-Tg mice with a combination therapy of CEA/TRICOM vaccine and ^90^Y-labeled anti-CEA bearing mAb in MC38 mice. Overall survival was improved compared to vaccine or ^90^Y-labeled anti-CEA mAb only, with increased tumor-infiltrating CEA-specific CD8+ T cells and IFN-γ 81.

Although some clinical trials have yielded promising results, others have shown no clear survival benefit from particular combination treatments. 3.7 MBq ^177^Lu-DOTA-diZD combined with anti-PD1 mAb treatment did not improve the survival of mice with TNBC 95.

See Table 3-5 for a summary of pre-clinical studies, clinical study, and retrospective study of a combination strategy.

## Challenges and perspectives

In summary, although still anecdotal, evidence is emerging to support the concept that local radiation therapy and immuno-therapy can successfully synergize and produce a therapeutically effective antitumor immune response, even in metastatic cancer. It is the very beginning of a novel field. More research is warranted to define the many mechanisms underlying the crosstalk with the immune system and to establish how best to harness ionizing radiation in this new role.

Whether as a direct inducer of immunogenic cell death or in its application as a simple adjuvant to more complex immunotherapy manipulations, radiation therapy is once again playing a central role in the management of cancer at any stage and the ever-lasting quest for cancer cure. However, due to the limited understanding of the phenomenon and mechanism of RPT-induced anti-tumor immunity, and the existing research has continued the same direction as traditional radiotherapy, the similarities and uniqueness of RPT compared with traditional radiotherapy in immunity are still unknown, and need further exploration.

Aside from this, for the combination of RPT and IT, improved preclinical model systems for RPT-dose definition are needed, as well as a detailed and easy-to-perform immune monitoring of patients. The aims of innovative RPT in multimodal radioimmunotherapy concepts are diverse: assure local tumor control, stop the proliferation of tumor cells, induce tumor shrinkage, induce tumor cell death, alter the tumor cell phenotype, induce ICD together with an immune stimulatory microenvironment, foster infiltration of immune cells into the tumor with consecutive activation of the latter, converse immune suppressive conditions in activation ones, generate increased tumor antigen pool and neoantigens, induce specific and long-lasting anti-tumor immune responses against the primary tumor and metastases. But these are not mere wishes; every single aim of traditional radiotherapy is already achievable under distinct conditions. The big challenge will be to understand these conditions induced by RPT and to exploit them for a very personalized combination strategy in the future.

Even though extensive research is carried out in the field of radiation oncology, most clinical studies only consider the effects of radiation on the local tumor tissue. The influence of dose, fractionation and timing particularly about immune activation is not been satisfactorily investigated so far. However, this is of particular interest, since recent studies, including additive immune therapy approaches, showed that not every therapy combination of classical RPT concepts and IT is equally successful, the heterogeneous influences on the immune system of today's RPT schemes need more attention.

Though some evidence on PRT-induced immune activation, particularly in preclinical mouse models, have been reporte, differences in the doses and duration may lead to discordant immune activation between preclinical and clinical exploration. In the case of ^177^Lu-DOTATATE, treatment of mice with 30-40 MBq (1500-2000 MBq/kg for 20 g mice, single dose) caused increased infiltration of CD86+ APC and FasL-expressing NK cells 93, yet the clinical use of 7.4 GBq (92.5 MBq/kg for 80 kg patients, 4 doses every 8 weeks) may cause neutropenia, thrombocytopenia, and lymphocytopenia (1%, 2%, and 9% of patients in the ^177^Lu-DOTATATE group, respectively) 123. Although these hematological events were transient, the hematological toxicity caused by RPT remains unknown whether it inhibits the activation process of immunity.

Beyond this, the long-term adverse events to immune cells are often unpredictable. In clinical practice, bone marrow is the critical target, with the reduced bone marrow reserve, and more infrequently, myelodysplastic syndrome (MDS) which was observed in approximately 2% of patients treated with ^177^Lu-DOTATATE 124. Even though the overall incidence of PRRT-induced myelosuppression is acceptable, it suggests to us that a complex relationship between RPT procedure and immune activation exists that requires more experimental evidence.

Reducing or avoiding damage to the immune cells and bone marrow from RPT may allow for a more rapid response of immunity. Therefore, it is of great interest to explore the interaction between short-term hematologic toxicity and immunity, the mechanisms of long-term toxicity, and methods to protect immune system. Up to now, chemical perturbing tools based on radiotherapy and PET probes have been developed to regulate biological effects 125-130, which may potentiate RPT by balancing toxicity and efficacy via RPT-mediated controlled drug release in vivo.

To this end, combined, multi-modal treatment regimens have to be developed with the capacity to induce immunogenic forms of tumor cell death and concomitantly activate the immune system. The close collaboration of clinical radiation oncologists, surgeons, radiobiologists, molecular oncologists, and immunologists is indispensable to develop and optimize the personalized therapeutic regime with the highest benefit for each individual patient.

## Figures and Tables

**Table 1:** The influence on anti-tumor immune stimulation in pre-clinical in vitro and in vivo models.

Radionuclide	t 1/2	Range (mm)	Drug	Model (tumor cell line or indication)	Immune response	Ref
**α**						
^213^Bi	45.6 min	0.05	^213^Bi-BSA	Mouse vaccine (MC38)	T cells are essential for the antitumor effectDAMPs (Hsp70 and HMGB1) ↑DCs ↑	66
^223^Ra	11.4 d	0.05-0.08	Xofigo	Cell (LNCaP, PC3, MDA-MB-231, ZR75-1, H441, H1703)	MHC-I and calreticulin ↑CD8+ T cell cytotoxic efficiency specific for MUC-1, brachyury, and CEA tumor antigens ↑	72
			Xofigo	Human (Prostate cancer)	PD-1 expressing EM CD8+ T cells ↓CD8+ T cells and their subsets (N.S.)CD27, CD28 or CTLA4-expressing T cells (N.S.)CD8+ T cells producing IFN-γ, TNF-α and IL-13 (N.S.)	73
			-	Human (Prostate cancer)	T cells that expressed ICOS, TIM-3, PD-L1, and PD-1 ↑Treg cells and MDSC ↑	74
			-	Human (Prostate cancer)	Abscopal effect IL-6 ↑	75
^227^Th	18.7 d		BAY 2287411	Cell (OVCAR-3)	DAMP markers (calreticulin, HSP70, HSP90, and HMGB1) ↑	76
^211^At	7.2 h	0.05	[^211^At]MM4	Mouse (GL26)	Tumor-associated macrophages ↑CD4+ T cells ↑	77
			^211^At-ATE-MnO_2_-BSA	Mouse (4T1, CT26)	CD80+CD86+ DCs ↑	78
**β**						
^90^Y	64.1 h	5.3	^90^Y-labeled anti-CEA mAb	Mouse (MC38-CEA+)	Fas ↑	81
			^90^Y-NM600	Cell (B78, MOC2)	IFNγ ↑ CTLA-4 of T cells ↑	83
			^90^Y-NM600	Mouse (EL4)	CD8+ T ↑ Treg cells ↓	82
			^90^Y-NM600	Mouse (B78)	CD11b+ and NK cells ↑ Effector T cells (CD8+) / suppressor Tregs (CD4+CD25+FOXP3+) ↑ Vcam1, Fas and IFNβ ↑ cGAS/STING pathway activation was required for antitumor efficacy	84
			^90^Y radioembolization	Human (Colorectal cancer)	low levels of tumor-infiltrating lymphocyte (TIL) infiltration in tumor cancer islands before and after Y90 radioembolization (N.S.)	85
^131^I	8 d	0.8	-	Human (Thyroid cancer)	Th17, Tc17 and Treg cells increased ↑IL-17, IL-23, IL-10 and TGF-β1 ↑	88
			^131^I-Cat-CpG/ALG	Mouse (4T1, CT26)	Effector memory T cells ↑ TNF-α and IFN-γ ↑	89
^153^Sm	46.5 h	0.4	^153^Sm-EDTMP	Cell (LNCaP)	Surface molecules (Fas, CEA, MUC-1, MHC class I, and ICAM-1) ↑ Antigen-specific CTL-mediated killing ↑	91
^177^Lu	6.6 d	0.62	^177^Lu-DOTATATE	Mouse (NCI-H727)	FasL+ CD49b+ NK cells ↑	93
			^177^Lu-EB-RGD	Mouse (MC38)	CD45+/PD-L1+ and cd11b+/PDL1+ cells ↑	94
			^177^Lu-DOTA-diZD	Mouse (4T1)	CD4+ and CD8+ cells ↑	95
^188^Re	16.9 h	3.1	^188^Re-6D2	Mouse (A2058)	Complement C3 ↑	96
^18^F	109.7 min	2.39	2-[^18^F]FDG	Cell/Mouse (MC38, CT26)	PD-L1 ↑	97
^64^Cu	12.7 h	2.5	^64^Cu-DOTA-EB-cRGDfK	Cell (MC38, CT26)	PD-L1 ↑	99

DAMPs: Damage-associated molecular patterns; DCs: Dendritic cells; MHC-1: Major histocompability complex-1; MUC-1: mucin-1; CEA: Carcinoma embryonic antigen; N.S.: No significance; MDSC: Myeloid-derived suppressor cell.

**Table 2:** Summary of described immune effects by RPT.

Classification	Description	Model (tumor cell line or indication)	Radionuclide	Ref
DAMP	Calreticulin, HSP70, HSP90, and HMGB1 ↑	Cells (MC38, MDA-MB-231, ZR75-1, LNCaP, PC3, H1703, H441, OVCAR-3)	^223^Ra ^227^Th ^213^Bi	66, 72, 76
Tumor surface	Fas, ICAM-1 ↑	Cells (MC38-CEA+)/Mouse (B78)	^153^Sm ^90^Y	81, 84
	MHC-I ↑	Cells (MC38, MDA-MB-231, ZR75-1, LNCaP, PC3, H1703, H441, OVCAR-3)	^233^Ra ^153^Sm	72, 91
	Other TAAs (PSA, CEA and MUC-1) ↑	Cells (LNCaP)	^153^Sm	91
	PD-L1 ↑	Cells/Mouse (MC38, CT26)	^18^F ^64^Cu	97, 99
T cells	T cells are essential for the antitumor effect	Mouse (MC38)	^213^Bi	66
	CTL ↑	Mouse (EL4)	^90^Y	82
	T cell cytotoxic efficiency ↑	Cells (MC38, MDA-MB-231, ZR75-1, LNCaP, PC3, H1703, H441, OVCAR-3)/Mouse (MC38)	^213^Bi ^223^Ra	66, 72
	Costimulatory molecules (ICOS and CTLA-4) ↑	Cells (MC38-CEA+)/Human (Prostate cancer)	^223^Ra ^90^Y	74, 81
	Effector memory T cells ↑	Mouse (4T1, CT26)	^131^I	89
	Th17 and Tc17 ↑	Human (Thyroid cancer)	^131^I	88
	CTL/Treg ↑	Mouse (B78)	^90^Y	84
	Treg ↓	Mouse (EL4)	^90^Y	82
	Treg ↑	Human (Prostate cancer, thyroid cancer)	^223^Ra ^131^I	74, 88
	Inhibitory molecules (PD-1) ↓	Human (Prostate cancer)	^223^Ra	73
	inhibitory molecules (PD-1 and Tim-3) ↑	Mouse (MC38)/Human (Prostate cancer)	^223^Ra ^177^Lu	74, 94
Cytokine	IFNγ, IFNβ, IL-17, IL-23, IL-10, TGF-β1, IL-6, CXCL-10↑	Mouse (B78, MOC2, 4T1, CT26)/Human (Thyroid cancer)	^90^Y ^131^I	83, 88, 89
Other immune cells	DC, Innate myeloid, NK cells, and MDSC ↑	Mouse (MC38, B78)/Human (Prostate cancer)	^213^Bi ^90^Y^ 223^Ra	66, 74, 84
Complement	Complement C3 ↑	Mouse (A2058)	^188^Re	96
cGAS/STING	cGAS/STING ↑	Mouse (B78)	^90^Y	84

MHC-1: Major histocompability complex-1; TAAs: Tumor-associated antigens; PSA: Prostate-specific antigen; CEA: Carcinoma embryonic antigen; MUC-1: mucin-1; CTL: cytotoxic T lymphocyte; DC: Dendritic cell; NK: Natural killer; MDSC: Myeloid-derived suppressor cell.

**Table 3:** Pre-clinical studies of combination strategy

Radiopharmaceutical	Dose	Combination strategy	Indication	Therapeutic outcome	Immune response	Ref
**↑**						
^225^Ac-PSMA-617	30 kBq	Anti-PD-1 mAb	Prostate cancer	Reduces tumor burdenImproves TTP and survival	-	115
[^211^At]MM4	36 MBq/kg	Anti-PD-1 mAb	Glioma	Decreasing tumor burden	-	77
^213^Bi-anti-hPD-L1 mAb	125 kBq/g	-	Melanoma	Increased tumor growth delay	-	120
^177^Lu-DOTA-Y003	11.1 MBq	-	Colon cancer	Improve anti-tumor efficacyProlong overall survival	-	121
^131^I-αPD-L1	11.1 MBq	Anti-PD-L1 mAb	Colon cancer	Improve anti-tumor efficacyProlong overall survival	-	122
^177^Lu-DOTA-folate	5 MBq	Anti-CTLA-4 mAb	Breast tumor	Improve anti-tumor efficacyProlong overall survival	-	116
^177^Lu-DOTA-PEG4-LLP2A	30 MBq	Anti-PD-L1 mAb, anti-PD-1 mAb, anti-CTLA-4 mAb	Melanoma	Improved overall survival	-	119
^211^At-ATE-MnO_2_-BSA	15 μCi	Anti-PD-L1 mAb	Colon cancer	Improve anti-tumor efficacyProlong overall survival	CD8+ T cells infiltration, effector memory CD4+ T and CD8+ T cells, TNF-α, IFN-γ ↑	78
2-[^18^F]FDG	37 MBq	Anti-PD-L1 mAb	Colon cancer	Improve anti-tumor efficacyProlong overall survival	Effector memory T cells, CD4+ Th1, CD8+ TCLs, M1-like macrophages infiltration ↑ Tregs, M2-like macrophages infiltration↓ TNFa, IFNγ, and IL6 ↑	97
^64^Cu-DOTA-EB-cRGDfK	18.5 MBq	Anti-PD-L1 mAb	Colon cancer	Improve anti-tumor efficacyProlong overall survival	CD8+ CTLs and CD4+ Th1 cells infiltration, CD8+ CTL/Treg, CD4+ Teff/Treg, TEM cells ↑	99
^177^Lu-EB-RGD	18.5 MBq	Anti-PD-L1 mAb	Colon cancer	Improve anti-tumor efficacyProlong overall survival	CD8+ T cells infiltration ↑	94
^90^Y-NM600	7 MBq	Anti-CTLA-4 mAb	Melanoma	Improved overall survival	CD45+ immune cells, CD3+ T cells, CD8+ effector T cells, CD8+CD103+ tissue resident effector memory T cells, and γδ T cells ↑ PD-1 ↓IL-10 ↓Clonal expansion of T cells ↑IFNγ ↑	84
^131^I-Cat/ALG hybrid solution	50 μCi	Immune-adjuvant CpG, anti-CTLA-4 mAb	Breast cancer	Long-term immune memory protectionAbscopal effectInhibited tumor metastases	CD8+ CTL infiltration in the distant tumors ↑ Treg in the distant tumors ↓The CTL/Treg ratio ↑TNF-α and IFN-γ ↑Both CD4+ and CD8+ T cells are important	89
^90^Y-labeled anti-CEA mAb	6 MBq	CEA/TRICOM vaccine	Colon cancer	Improved overall survival	Tumor-infiltrating CEA-specific CD8+ T cells ↑IFN-γ ↑	81
**N.S.**						
177Lu-DOTA-diZD	3.7 MBq	Anti-PD1 mAb	TNBC	Not improve the survival	-	95

TTP: Time to progression; MDSCs: Myeloid-derived suppressor cell; CTL: cytotoxic T lymphocyte; CEA: Carcinoma embryonic antigen.

**Table 4:** Clinical studies of combination strategy

Radiopharmaceutical	Combine drug	Clinical trail	Indication	Phase	Estimated/ completion date	Participants
**Complete**						
^90^Y glass microspheres	Ipilimumab	NCT01730157	Uveal melanoma with liver metastases	Phase 1	Feb-16	6
^153^Sm-EDTMP	PSA-TRICOM	NCT00450619	mCRPC	Phase 2	Jan-17	44
^223^Ra, Xofigo	Sipuleucel-T	NCT02463799	Asymptomatic or minimally symptomatic bone-mCRPC	Phase 2	Dec-19	32
^223^Ra dichloride	Atezolizumab	NCT02814669	mCRPC	Phase 1	Jul-19	45
^177^Lu-DOTATATE	Nivolumab	NCT03325816	Extensive-stage small cell lung cancer	Phase I /2	Aug-20	9
**Ongoing**						
^177^Lu-PSMA-617	Pembrolizumab	NCT03805594	mCRPC	phase Ib	Apr-24	16
^177^Lu-PSMA	Pembrolizumab	NCT03658447	Prostate cancer	Phase I/2	Dec-22	37
^177^Lu-DOTATATE	Avelumab	NCT04261855	Merkel cell carcinoma	Phase I/2	Jan-24	65
^223^Ra, Xofigo	Pembrolizumab	NCT03996473	NSCLC with bone metastases	Phase I/2	May-23	164
^223^Ra	Pembrolizumab	NCT03093428	mCRPC	Phase 2	Jun-24	45
^223^Ra dichloride	Avelumab	NCT04071236	Advanced prostate cancer	Phase I/2	Jan-23	24
^223^Ra	Nivolumab	NCT04109729	mCRPC	Phase I/2	Jun-24	36
^225^Ac-J591	Pembrolizumab	NCT04946370	mCRPC	Phase I/2	Jun-28	76
^90^Y radioembolization, ^177^Lu-DOTATATE	Pembrolizumab	NCT03457948	Neuroendocrine tumors and liver metastases	Phase 2	Mar-24	32
^90^Y radioembolization	Nivolumab	NCT03033446	Hepatocellular carcinoma	Phase I/2	Dec-22	40
^90^Y glass microspheres	Nivolumab	NCT02837029	Advanced liver cancer	Phase 1	Jul-23	35
^90^Y radioembolization	Durvalumab	NCT04108481	Metastatic colorectal cancer	Phase I/2	Dec-25	18
^90^Y radioembolization	Pembrolizumab	NCT03099564	Hepatocellular carcinoma	Phase 1	Jan-23	30
^90^Y radioembolization	Ipilimumab, Nivolumab	NCT02913417	Uveal melanoma with liver metastases	Phase I/2	Jun-23	26
^131^I	Durvalumab	NCT03215095	Recurrent/metastatic thyroid cancers	Phase 1	Jul-23	11

mCRPC: Metastatic castrate-resistant prostate cancer; NSCLC: Non-small cell lung cancer.

**Table 5:** Clinical trial and retrospective study of combination strategy

Radiopharmaceutical	Combine drugs	Indication	Phase/ patients	Therapeutic outcomes	Ref
**Clinical Trial**					
^177^Lu-PSMA-617	Pembrolizumab	mCRPC	phase Ib	Durable responses in a subset of mCRPC without high mutational burden or microsatellite instability, suggesed a possible immunogenic priming effect of radioligand therapy.	133
^223^Ra dichloride	Atezolizumab	mCRPC	Phase 1	This Phase 1b study did not seem to show clinical benefit from combination.	134
^177^Lu-PSMA	Pembrolizumab	mCRPC	Phase I/2	The combination of anti-PD-1 and RNT synergistically reduces tumour burden and improves the time to progression and overall survival.	135
^177^Lu- DOTATATE	Nivolumab	Neuroendocrine tumors of the lung	Phase 1	The combination was well tolerated with most TRAEs with initial signs of antitumor activity.	136
^153^Sm-EDTMP	PSA-TRICOM	mCRPC	Phase 2	The primary endpoint was the proportion of patients without radiographic disease progression at 4 months. There was no statistical difference in the primary endpoint.	137
^223^Ra, Xofigo	sipuleucel-T	Bone- mCRPC	Phase 2	Patients in the combination arm were more likely to have a >50% PSA decline, longer PFS and OS, but the paradoxically lower immune responses observed.	138
^90^Y radioembolization	DurvalumabTremelimumab	MSS CRC	Phase 2	Limited benefits of radiation on promoting antitumor immune response in MSS CRC.	85
**Retrospective study**					
^177^Lu-PSMA	Pembrolizumab	mCRPC	1 patient	The combination strategy might be well tolerated in single patients.	139
^90^Y radioembolization	Nivolumab	Advanced HCC	1 patient	The combination served to increase their response rate and depth of response in HCC.	140
^90^Y radioembolization	NivolumabIpilimumab	HCC	26 patients	The combination strategy appeared safe, with no incidence of early toxicity or mortality in HCC patients.	141
^90^Y radioembolization	Pembrolizumab Ipilimumab Nivolumab	Unresectable hepatic metastases from UM	11 patients	The combination strategy is safe and effective and may improve hPFS and OS in patients with hepatic metastases from UM.	142

mCRPC: Metastatic castrate-resistant prostate cancer; TRAEs: Treatment-related adverse events; MSS: Microsatellite stable metastatic; CRC: Colorectal cancer; HCC: Hepatocellular carcinoma; PSA: Prostate-specific antigen; PFS: Progression-free survival; OS: Overall survival; UM: Uveal melanoma.

## Abbreviations

RPT: radiopharmaceutical therapy
ICD: immunogenic cell death
TME: tumor microenvironment
DAMPs: danger-associated molecular patterns
IT: immunotherapy
DDR: DNA damage response
TAAs: tumor-associated antigens
APCs: antigen-presenting cells
cGAS-STING: cyclic GMP-AMP synthase-stimulator of interferon genes
HMGB1: high mobility group box 1
DCs: dendritic cells
TLRs: Toll-like receptors
NKs: natural killer cells
CTLs: cytotoxic T lymphocytes
TNF-α: tumor necrosis factor-α
Hsp70: heat shock protein 70
mCRPC: metastatic castration-resistant prostate cancer
PBMCs: peripheral blood mononuclear cells
TEM: effector memory T cells
ICOS: expressed costimulatory
TIL: tumor‐infiltrating lymphocyte
DTC: differentiated thyroid cancer
GEP-NETs: gastroenteropancreatic neuroendocrine tumors
PRRT: peptide receptor radionuclide therapy
PET: positron emission tomography
ICI: immune checkpoint inhibitors
PD-1: programmed death 1
CTLA-4: cytotoxic T-lymphocyte-associated antigen 4
Treg: regulatory T cells
MDS: myelodysplastic syndrome
MHC-1: Major histocompability complex-1
MUC-1: mucin-1
CEA: Carcinoma embryonic antigen
MDSC: Myeloid-derived suppressor cell
